# Histopathological and Immunohistochemical Prognostic Factors in High-Grade Non-Endometrioid Carcinomas of the Endometrium (HG-NECs): Is It Possible to Identify Subgroups at Increased Risk?

**DOI:** 10.3390/diagnostics13132171

**Published:** 2023-06-26

**Authors:** Michele Paudice, Chiara Maria Biatta, Giulia Scaglione, Alessia Parodi, Serafina Mammoliti, Melita Moioli, Maria Grazia Centurioni, Fabio Barra, Simone Ferrero, Franco De Cian, Katia Mazzocco, Valerio Gaetano Vellone

**Affiliations:** 1 Department of Integrated Diagnostic and Surgical Sciences (DISC), University of Genoa, 16100 Genoa, Italy; michele.paudice@unige.it (M.P.); alessiaparodi20@gmail.com (A.P.); decianet@unige.it (F.D.C.); 2 Pathology University Unit, IRCCS Ospedale Policlinico San Martino, 16132 Genoa, Italy; 3 Anatomy and Pathological Histology, Veneto Institute of Oncology IOV-IRCCS, 35128 Padua, Italy; chiara.biatta@gmail.com; 4 Pathology Unit, Fondazione Policlinico A. Gemelli IRCCS, 00168 Rome, Italy; scaglione.giulia90@gmail.com; 5 Oncology Unit 1, IRCCS Ospedale Policlinico San Martino, 16132 Genoa, Italy; serafina.mammoliti@hsanmartino.it; 6 Obstetrics & Gynecology University Unit, IRCCS Ospedale Policlinico San Martino, 16132 Genoa, Italy; melita.moioli@hsanmartino.it (M.M.); simoneferrero@me.com (S.F.); 7 Obstetrics & Gynecology, IRCCS Ospedale Policlinico San Martino, 16132 Genoa, Italy; mariagraziacenturioni@hsanmartino.it; 8 Department of Health Sciences (DISSAL), University of Genoa, 16132 Genoa, Italy; fabio.barra@icloud.com; 9 Department of Neurosciences, Rehabilitation, Ophthalmology, Genetics, Maternal and Child Health (DINOGMI), University of Genoa, 16132 Genoa, Italy; 10 General Surgery University Unit, IRCCS Ospedale Policlinico San Martino, 16132 Genoa, Italy; 11 Pathology Unit, IRCCS Istituto Giannina Gaslini, 16132 Genoa, Italy; katiamazzocco@gaslini.org

**Keywords:** endometrial carcinoma, immunohistochemistry, prognosis

## Abstract

Endometrial cancer is an emerging disease with an increase in prevalence of aggressive histotypes in recent years. Background: In the present study, potential histopathological and immunohistochemical prognostic markers were investigated. Consecutive cases of high-grade non-endometrioid carcinoma (HG-NEC) of the endometrium were considered. Methods: Each surgical specimen was routinely processed; the most significant block was selected for immunohistochemistry and tested for ER, PR, ki67, p53, E-cadherin, β-catenin, Bcl-2 and cyclin D1. For each immunomarker, the percentage of positive tumor cells was evaluated (%) and dichotomized as low and high according to the distribution in the study population. Follow-up was collected for disease-free survival (DFS) and overall survival (OS). Thirty-three cases were eligible: 19 resulted in FIGO I–II; 14 resulted in FIGO III–IV. Twelve patients suffered a recurrent disease (mean follow-up 24.6 months); 8 patients died of the disease (mean follow-up 26.6 months). Results: Women with recurrent disease demonstrated a significantly higher Bcl2% (35.84 ± 30.96% vs. 8.09 ± 11.56%; *p* = 0.0032) while DOD patients had higher ki67% (75 ± 13.09% vs. 58.6 ± 19.97%; *p* = 0.033) and Bcl2% of border significance (34.37 ± 34.99% vs. 13 ± 17.97%; *p* = 0.078). As expected, FIGO III–IV had a worse DFS (HR = 3.34; 95% CI: 1.1–10.99; *p* = 0.034) and OS (HR = 5.19; 95% CI: 1.27–21.14; *p* = 0.0217). Bcl-2-high patients (Bcl2 > 10%) demonstrated a significantly worse DFS (HR = 9.11; 95% CI: 2.6–32.4; *p* = 0.0006) and OS (HR = 7.63; 95% CI: 1.7–34; *p* = 0.0084); moreover, PR low patients (PR ≤ 10%) had significantly worse DFS (HR = 3.74; 95% CI: 1.2–11.9; *p* = 0.02). Conclusions: HG-NEC represents a heterogeneous group of endometrial aggressive neoplasms with a worrisome prognosis, often at an advanced stage at presentation. Bcl-2 and PR may represent promising markers to identify a subgroup of patients having an even worse prognosis requiring a careful and close follow-up.

## 1. Introduction

Endometrial carcinoma (EC) is a very common neoplasm among women, being the sixth cause of cancer in the world, and the fourth in the USA and in Italy (5% of all tumors). It counts for about 320.000 new cases and 76.000 deaths per year [1]. Its incidence is higher and still increasing in Western industrialized countries due to the higher incidence of its risk factors and the longevity of the population [2]. In Italy, it is estimated that 1 in every 47 women will develop EC in her life [3].

Seventy-five percent of EC cases are diagnosed in women older than 50 years old [4]. It also appears that, as the age of diagnosis increases, so does tumor aggressiveness, with more frequent TP53 mutations and E-cadherin loss of expression [5].

EC has been long categorized into two major classes, based on clinical–pathological correlations: type I and type II carcinoma [6]. EC type I, or endometrioid EC, represents the majority of sporadic endometrial carcinomas (70–80%). It is a moderately indolent tumor that generates after prolonged estrogenic stimulation. EC type II, or non-endometrioid EC, is less frequent (about 10–20% of endometrial carcinomas) but more aggressive and usually not related to estrogen excess or to endometrial hyperplasia. They are typically high-grade carcinomas and include non-endometrioid differentiation, most frequently serous, less frequently clear cell, mixed or undifferentiated [7].

In this context, high-grade non-endometrioid endometrial carcinomas (HG-NECs) constitute the histopathologic manifestation of type II carcinomas.

Since Bockman’s classification, numerous molecular studies on endometrial cancer have been carried out and dozens of molecular markers have been proposed over the years as prognostic markers. TCGA (Cancer Genome Atlas Research Network) has performed genomic, transcriptomic and proteomic characterization of EC using the most modern array and sequencing-based technologies. As a result, a new classification dividing ECs into four classes has been proposed, representing the future in endometrial carcinoma research and therapy [8]. Recently, the ESMO/ESGO/ESP guidelines proposed a prognostic stratification based on few immunohistochemical markers and on POLE sequencing [9].

However, in particular, POLE sequencing is not possible in all centers and the aforementioned guidelines provide for prognostic stratification even without molecular characterization.

In the current study, we chose a small panel of molecules valued with immunohistochemistry, previously proposed as prognostic markers in EC, commonly used in daily practice and available in many labs worldwide.

Overall, steroid hormones (mainly estrogen and progesterone) have been considered as playing a key role in the pathogenesis of EC, especially in type I carcinoma. Estrogen (ER) and progesterone receptors (PR) are able to influence prognosis and clinical management as well, as they correlate with grading and staging [10].

ERs are expressed in 60–70% of ECs. They have a pivotal role in the carcinogenesis of type I tumors [11]. Conditions resulting in long-lasting unopposed exposure to estrogen (obesity, exogenous hormone replacement therapy, polycystic ovary syndrome, anovulation and type 1/2 diabetes mellitus) can promote the development of atypical endometrial hyperplasia and increase the risk of EC [12]. The loss of ERα and PR has been correlated with poor survival, whereas expression of ERβ has not shown any clinical pathological correlation [10]. The loss of ERα is associated with high-grade tumors. In contrast, ERα expression is related to low-grade and low stage of disease.

Progesterone is the physiological estrogen antagonist [13]. It acts by decreasing the risk of developing estrogen-related cancer through several mechanisms, such as reduction in ER and increase in the metabolic inactivation of estrogen. Thus, estrogen-related endometrial hyperplasia can be treated using progestin therapy [14].

Ki67 is a nuclear antigen expressed by proliferating cells (phases G1, S, G2, mitosis), but absent in resting cells (G0). High ki67 expression is related with a more aggressive behavior of cancer [15].

The TP53 oncosuppressor gene (chromosome 17) encodes p53 nuclear protein, a transcriptional factor involved in cell cycle arrest and apoptosis. After DNA damage, p53 accumulates and stops the cell cycle through inhibition of cyclin D1 phosphorylation and, if necessary, by promoting apoptosis through interaction with Bax and Apaf-1 proteins. TP53 mutations are typical of EC type II, in particular of serous carcinoma [16]. The majority of TP53 mutations are missense and lead to the loss of oncosuppressor function. In normal cells, p53 is rapidly destroyed and cannot be seen using immunohistochemistry (IHC). Missense mutations are clearly visible using IHC because there is nuclear accumulation of aberrant p53: the most common IHC pattern is widespread and intense nuclear positivity [17]. The recent ESMO/ESGO/ESP has a crucial role in prognostic stratification [9].

β-catenin is encoded by the CTNNB1 gene (chromosome 3), and the protein mediates the link between actin filaments of the cytoskeleton and transmembrane E-cadherin. The IHC nuclear accumulation of β-catenin due to gene mutation is significantly more common in EC type I (31–47%) if compared with EC type II (0–3%). On the contrary, E-cadherin mutation is more frequent in EC type II. Usually, EC type I with CTNNB1 mutation has favorable prognosis and low stage [18].

E-cadherin is encoded by the CDH1 gene (chromosome 16) and constitutes another adhesion molecule, essential for tight junctions between cells. These molecules mediate the connection between cells through a calcium-dependent mechanism [19]. CDH1 is considered an oncosuppressor gene because it controls cell cohesiveness. Low E-cadherin expression is related to major tumor cell exfoliation and high risk ok peritoneal metastasis. E-cadherin mutation is present in 60% of EC type II and in 22% of EC type I, where it is associated with more aggressive behavior [20]. The partial or total loss of E-cadherin is reported to be associated with adverse prognosis and short survival.

Bcl-2 is a protein with antiapoptotic activity that was identified for the first time in non-Hodgkin’s follicular lymphoma. Bcl-2 expression is correlated with many human cancers, including kidney and prostate cancers, thyroid cancer and non-small cell lung cancer. Loss of Bcl-2 is associated with independent negative prognostic factors, such as a greater depth of myometrial invasion, aggressive histotype, loss of expression of PR, and advanced FIGO stage at diagnosis. Other studies showed a correlation between loss of Bcl-2 and risk of lymph node metastasis and recurrence [21,22,23].

Cyclin D1 is encoded by CCND1, a protooncogene (chromosome 11). Its role is mainly pivotal in phase G of the cell cycle. Cyclin D1 mutation is more typical of EC type I [24]. Intracytoplasmic protein accumulation, detectable using IHC, has been related to an impairment of proteolytic degradation [25]. In EC, cyclin D1 overexpression has a negative prognostic value and is related with metastatic lymph node involvement [26]. Rarely, β-catenin and cyclin D1 are overexpressed together. Some studies showed that cyclin D1 alteration could be an early event in endometrial carcinogenesis; however, there is not much difference in its intensity of expression from hyperplasia to EC [27].

The aim of this study is to identify a subgroup of patients with HG-NECs having a worse prognosis in terms of disease-free survival (DFS) and overall survival (OS) using a limited panel of histopathological and immunohistochemical markers.

## 2. Materials and Methods

We retrospectively considered all patients treated with radical hysterectomy for endometrial carcinoma in our institution for the period 2013–2018. Only cases with a diagnosis of high-grade non-endometrioid carcinoma (HG-NEC) were included. Cases that had undergone neoadjuvant chemotherapy, previous hormonal therapy, with incomplete data or follow-up were excluded.

The hysterectomy specimens were routinely fixed and processed to obtain 3 µm-thick histological sections, finally stained with hematoxylin/eosin. Additional slides were cut from the most representative paraffin block and tested with a panel of IHC stains including ERα, PR, Ki67, p53, β-catenin, E-cadherin, Bcl-2 and cyclin D1. Histopathological examinations were reported using an institutional protocol.

For IHC, we used an automatic immunostainer, Benchmark XT (Ventana Medical Systems SA, Strasbourg, France). Antigen retrieval was performed using citrate buffer (pH 6) at 90 °C for 30 min, incubated in primary antibody for 1 h at 37 °C, followed by the addition of the polymeric detection system Ventana Medical System Ultraview Universal DAB Detection Kit, counterstained using modified Gill’s hematoxylin and mounted in Eukitt. The tested antibodies are described in Table 1.

For all the proposed molecular markers, the staining index (SI), accounting for the percentage (%) of positive tumor cells, was evaluated by two pathologists working separately and blind. Any discrepancy was discussed from a multiheaded microscope to a final decision.

On the basis of the distribution in the study population, the SIs of each proposed molecular marker were dichotomized in two discrete categories named low and high according to the cutoff values illustrated in Table 1.

For p53, a percentage of stain ≤ 10% was considered wild-type while a percentage > 10% was considered abnormal.

All patients were collectively discussed in a multidisciplinary disease management team (DMT) and treated according to guidelines [9], including follow-up.

The clinical, pathological and IHC data of the patients enrolled in the study were entered into a Microsoft Excel© spreadsheet.

Discrete variables were compared using the χ2 test; continuous variables were compared using a Kruskall–Wallis test. Correlations between continuous variables were evaluated using Spearman rank correlation. Survival univariate analysis was studied using Kaplan–Meier survival curves. For statistical computation, the MedCalc© program was used. In all cases, a degree of significance of 95% was chosen. In the tables, continuous numeric variables are expressed as mean ± standard deviation while continuous variables are expressed as the number of observed cases (percentage).

The current study was approved by the local ethical committee (CER Liguria 46/2020 DB id 10320).

## 3. Results

In the period considered for the purpose of this study, out of 252 EC patients, 46 had a diagnosis of HG-NEC (18.25%). A total of 33 cases were considered eligible for the aims of this study. Our study population was composed of elderly women (mean: 74.12 ± 15.53 years; minimum: 53; maximum: 93) who often had come to surgery at an advanced stage.

HG-NECs represent a heterogeneous group constituted by different histologic types. The histopathological examination of the surgical specimens frequently showed worrisome features such as infiltrative tumor borders, intratumoral necrosis and lymph–vascular space invasion. Less frequently, moderate/severe desmoplasia or moderate/severe tumor lymphocytic infiltrate were observed (Table 2).

The immunophenotyping of the neoplasms included in this study showed a fairly wide variability in stain index for all proposed molecular markers: ER and PR were generally low, such as in Bcl-2 and cyclin D1, while ki67, p53, β-catenin and E-cadherin resulted in being highly expressed (Table 3).

We observed a fairly strong and significant correlation between ER and PR staining indexes (rho = 0.716; *p* < 0.0001) and between ki67 and p53 (rho = 0.541 *p* = 0.0012) (Table 4).

During follow-up (mean DFS follow-up duration 24.84 ± 18.39 months; mean OS follow-up duration 27 ± 18.06 months), 12 patients (36.36%) recurred after surgery and 8 of them died of the disease (24.24%); as expectable, women with no recurrent disease had a longer follow-up (30.28 ± 19.86 months vs. 15.33 ± 10.59 months; *p* = 0.037).

**Figure 1:**
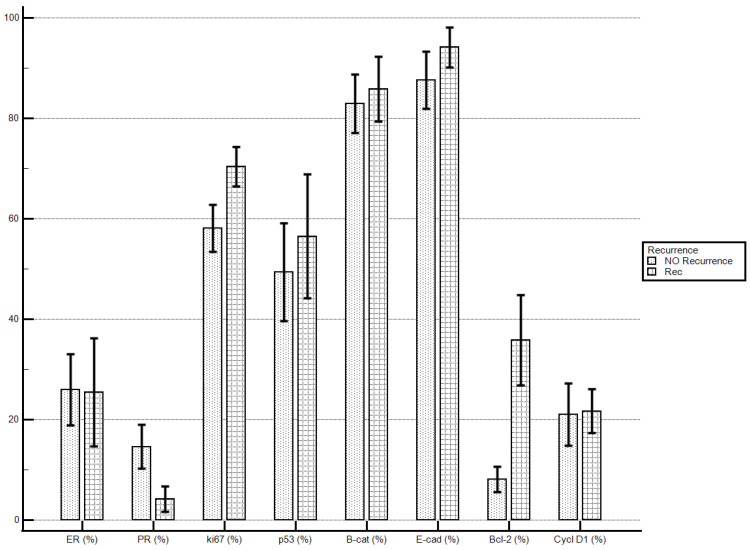
Disease recurrence and immunostaining percentage.

Considering the proposed markers’ staining index, women with recurrent disease demonstrated significantly higher levels of Bcl-2 if compared with patients with no recurrent disease (35.84 ± 30.96% vs. 8.09 ± 11.56%; *p* = 0.0032). No statistically significant differences were demonstrated for the other molecular markers’ staining indexes (Table 5; Figure 1).

**Figure 2:**
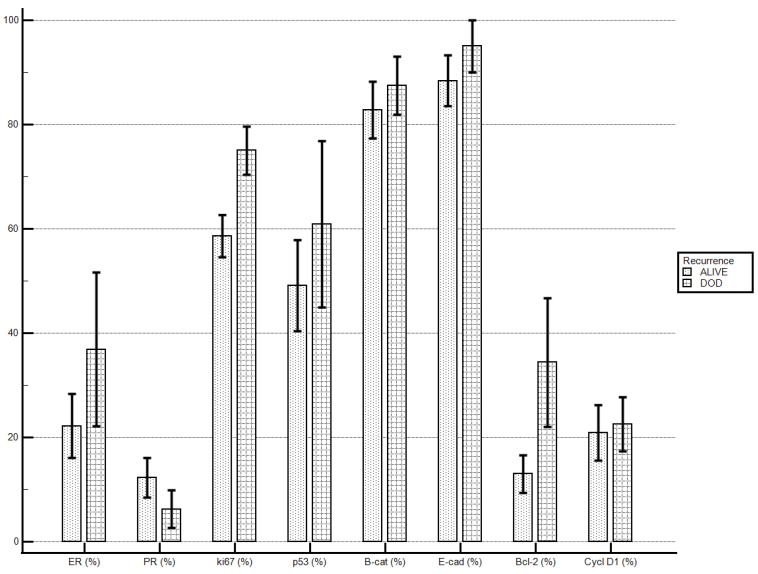
Death of the disease and immunostaining percentage.

Patients who died of the disease, if compared to patients alive at end of the follow-up, demonstrated higher levels of ki67 (75 ± 13.09% vs. 58.6 ± 19.97%; *p* = 0.033) and levels of Bcl-2 were tendentially higher (34.37 ± 34.99% vs. 13 ± 17.97%; *p* = 0.078). No statistically significant differences were demonstrated for the other molecular markers’ staining indexes (Table 6; Figure 2).

**Figure 3:**
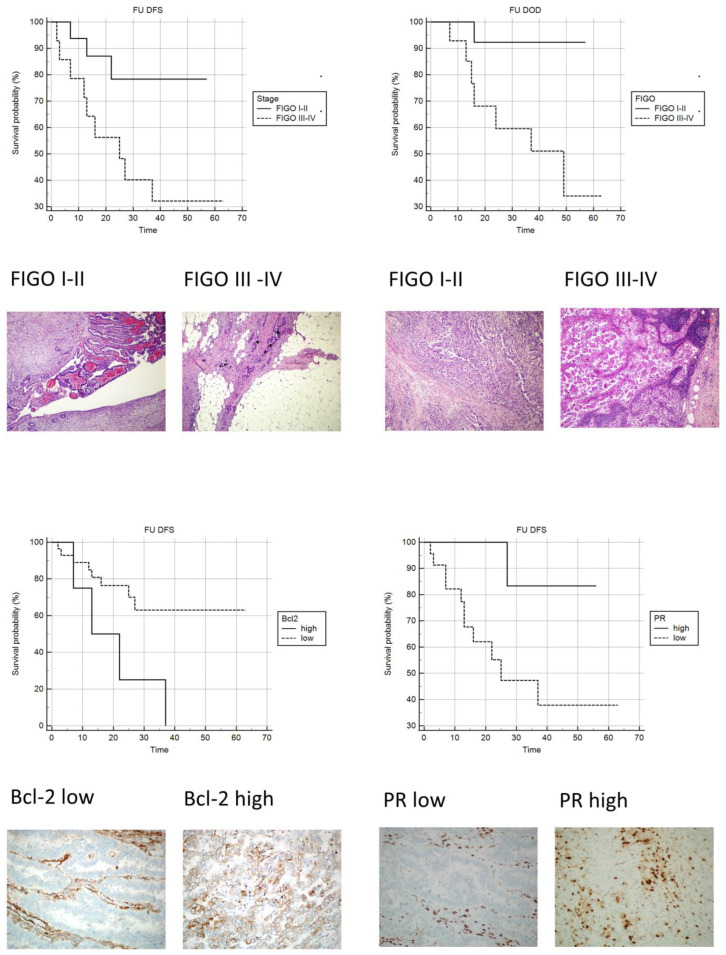
Kaplan-Meyer curves: **Upper-left**: DFS FIGO I–II vs. FIGO III–IV (original magnification 100×; H&E). **Upper-right**: OS FIGO III vs. FIGO IIIIV (original magnification 100×; H&E). **Lower-left**: DFS Bcl-2 low vs. Bcl-2 high (original magnification 100×; IHC stain Bcl-2). **Lower-right**: DFS PR low vs. PR high (original magnification 100×; IHC stain PR).

Upon univariate analysis, as expected, patients with metastatic disease at the time of surgery showed a significantly increased risk of both disease recurrence and dying of disease (Table 7; Figure 3).

An increased risk of recurrent disease was observed in patients with low PR and high Bcl-2 staining indexes (Table 7; Figure 3).

The other proposed histopathological and immunohistochemical markers failed to identify a statistically significant risk in terms of DFS or OS (Table 7 and Table 8).

## 4. Discussion

Endometrial carcinoma represents a disease growing in incidence, particularly in Western countries, paralleling the progressive ageing of the population and rising of known risk factors [28].

In particular, the rise in incidence of advanced stages and aggressive histologic types represents a matter of concern for endometrial cancer prevention and treatment.

Only with a better understanding of the molecular events underlying carcinogenesis in the various histotypes of endometrial cancer will it be possible to identify potential prognostic factors and individualized therapy targets.

The recently introduced TGCA classification represents a turning point in endometrial cancer comprehension. Nevertheless, after more than 30 years, the Bokhman and Kurman studies still remain pivotal and relevant. Type I of the traditional classification encompass cancers belonging to the first three classes of that classification, while, on the other hand, type II of the traditional classification encompass tumors of the fourth class. Class 4 tumors with a high number of copies are defined as “serous-like” and are characterized by a high number of aberrations in copy numbers and a low frequency of mutations. They seem to have peculiar mutations frequently involving TP53, FBXW7 and PPP2R1A genes. PTEN and KRAS mutations, typical of low-grade carcinomas with endometrioid histology instead, are rare. The prognosis of this group again appears unfavorable. This genomic class includes the majority of serous carcinomas, some mixed carcinomas and ¼ of endometrioid G3 carcinomas [29].

TGCA classification represents the future but requires advanced and expensive molecular techniques, currently available in few laboratories and, as a consequence, seldom used as a guide in a real-life clinical setting. Several groups are currently working on surrogate methods to incorporate TGCA findings into clinical practice [30].

Histopathological examination still represents the first line in daily diagnostics, and immunotesting is relatively inexpensive and well established worldwide: they currently represent the cornerstone of any clinical choice. In this context, HG-NECs require particular attention regarding the search for potential markers of aggressiveness and future targets for individualized therapies. Our series is small but representative of a heterogeneous group of relatively rare malignancies accumulated due to aggressive behavior and poor prognosis [31].

Comparing our results with the current literature was a challenging task; the results often appear contrasting, and very few studies are dedicated exclusively to non-endometrioid high-grade carcinomas. Many molecular and morphological prognostic factors have been proposed over the years, but it is well established how advanced stage and high grade are probably the most important factors affecting prognosis [32,33].

In many studies, an important bias is represented by grouping all high-grade endometrial carcinomas as a single entity, but high-grade endometrial carcinomas (HG-ECs) seem to have profound clinical–pathological and immunophenotype differences if compared to HG-NECs [34,35].

HG-NECs constitute approximately 20% of ECs and affect the elder population, frequently manifesting as an advanced stage of the disease. They often presented complex histology; more than half of them showed different histologic types; the largest subgroup was defined as mixed carcinoma, being composed of two epithelial components with at least one of them serous or clear cell. Another subgroup includes malignant mixed Müllerian tumor (MMMT), which is composed, by definition, of at least two components, one of which is epithelial and high grade. In both subgroups, the high-grade epithelial component was prominent, accounting for at least 30% of the entire tumor mass. We support the hypothesis that this high-grade epithelial component represents the “driving force” of the neoplasm. Pure neoplasms were rarer with incidences in line with the literature [36].

Although HG-NECs represent a heterogeneous group of tumors, they could be grouped by the common finding of worrisome features detectable both in hematoxylin/eosin and with the aid of immunohistochemistry.

The follow-up of the patients included in the study was quite heterogeneous, with a median duration of 22 months for DFS and 22 months for OS. HG-NECs were confirmed as being aggressive in nature, with patients rapidly relapsing and being driven to death by the disease.

Histopathological features, widely considered markers of aggressiveness, such as infiltrative tumor borders, intratumoral necrosis and lymph–vascular space invasion, were a common finding in our series, affecting the majority of the cases. Interestingly, other alarming features such moderate/severe desmoplasia or moderate/severe tumor lymphocytic infiltrate were observed in a minority of cases [32].

The wide variability observed in tumor cell morphology was paralleled in the proposed molecular markers’ staining indexes.

It is well established how ER is expressed in the majority of endometrial and breast carcinomas and its presence is associated with a less aggressive phenotype [37]; on the contrary, and as a general rule, HG-NECs show low levels of steroid hormone receptors, confirming their hormone insensitivity. PR, in contrast to ER, is suggested to be a more predictive factor of disease-free survival [10], and our findings confirm these observations.

The proliferation index ki67 evaluation resulted in being high, in some cases very high, in HG-NECs, confirming their aggressive behavior.

TP53 has a fundamental role in differentiating EC subgroups. The mutation of TP53 represents a crucial event in type II endometrial carcinoma carcinogenesis and progression. It is well reported how its accumulation represents a relevant prognostic factor [29]. As expected, in our study, population p53 staining index in general was high, failing to identify subgroups at increased risk of recurrence or dying of the disease. It should be noted that immunohistochemistry is able to detect only a part of TP53 mutations.

Even tested adhesion molecules β-catenin and E-cadherin failed to identify subgroups with increased risk. It was reported how low levels of these molecules are associated with metastatic deposition [17,38,39]. In our study population, more than half of the patients with low β-catenin and/or low E-cadherin had FIGO III–IV at surgery.

Bcl-2 is a protooncogene that exhibits antiapoptotic activity. Many regulators of the apoptotic process belong to the same family of Bcl-2, which consists of proteins that regulate the permeability of the outer mitochondrial membrane. Some of them have an antiapoptotic function, such as Bcl-2, Bcl-xl and Bcl-w, while others show proapoptotic activity, such as Bax, Bad, Bak and Bok [40,41].

Apoptosis is induced by the release of cytochrome c in the cytosol, with the subsequent activation of caspase 9 and caspase 3 [42]. A theory suggests that Rho proteins may have a role in the activation of Bcl-2, Bcl-1 and Bid. In fact, the inhibition of Rho decreases the expression of antiapoptotic proteins and increases the levels of the proapoptotic protein Bid. It also induces the release of caspase 9 and caspase 3 [17].

The immunohistochemical staining of Bcl-2 in the nonneoplastic endometrium has a strong variability; it increases in the proliferative phase and decreases in the secretory phase of the menstrual cycle. In these phases, Bcl-2 also plays an important role in regulating cell differentiation throughout the entire uterine cycle. Some studies have shown that the genes that regulate apoptosis may also be involved in the dysregulation of cell proliferation and death, the shift from simple to complex hyperplasia, and adenocarcinoma [41].

The loss of Bcl-2 is certainly associated with independent negative prognostic factors, such as deeper myometrial invasion, loss of PR expression, aggressive histotype and advanced FIGO stage. Other studies have shown a correlation between the loss of Bcl-2 and the risk of having lymph node metastases and recurrence [38].

In an old study, Athanassiadou demonstrated how, on in-print cytological specimens, Bcl-2 expression was associated with a good five-year survival. Interestingly, 18 cases of HG-NECs were also considered and none of them stained for Bcl-2 [39]. In another more recent study, Appel et al. failed to find any significant any correlation between Bcl-2 expression and histopathologic markers or survival. However, again, in this study, no distinction between the histologic type was attempted [23].

Our findings seem to confirm a prognostic role of primary importance for PR and specify the role of Bcl-2 in delimiting a group of patients at greater risk of recurrence. In conclusion, we can answer “yes” to the question in the title. The HG-NECs confirmed their clinical aggressiveness with frequently worrisome aspects. Although marked interindividual and intratumoral variability was observed, cases with advanced stage at surgery, low levels of PR and high levels of Bcl-2 showed a worse DFS. These patients could benefit from a close follow-up with thorough controls and more aggressive treatments.

## Figures and Tables

**Table 1:** List of antibodies.

Marker	Clone	Manufacturer	Dilution	Low SI	High SI
ER	6F11	Ventana	Prediluted	≤10%	>10%
PR	100	Ventana	Prediluted	≤10%	>10%
ki67	30-9	Ventana	Prediluted	<60%	≥60%
p53	DO-7	Ventana	Prediluted	≤10%	>10%
β-catenin	14	Ventana	Prediluted	<70%	≥70%
E-cadherin	36	Ventana	Prediluted	<70%	≥70%
Bcl-2	124	Ventana	Prediluted	<60%	≥60%
Cyclin D1	SP4-R	Ventana	Prediluted	<20%	≥20%

**Table 2:** Clinical–pathological and immunohistochemical features of the study population. Categorical variables.

HISTOTYPE	N	%
Mixed	13	39.39
Serous	9	27.27
MMMT	6	18.18
Undifferentiated	3	9.09
Clear Cell	2	6.06
FIGO staging	N	%
IA	10	30.30
IB	5	15.15
II	4	12.12
IIIA	2	6.06
IIIC1	6	18.18
IIIC2	5	15.15
IVB	1	3.03
STAGE	N	%
Local (FIGO I–II)	19	57.58
Metastatic (FIGO III–IV)	14	42.42
FOLLOW UP DFS	N	%
NO recurrence	21	63.64
Recurrence	12	36.36
FOLLOW UP OS	N	%
Alive	25	75.76
DOD	8	24.24
INVASION	N	%
Expansive	11	33.33
Infiltrative	22	66.67
DESMOPLASIA	N	%
Absent/Mild	24	72.73
Moderate/Severe	9	27.27
TIL	N	%
Absent/Mild	21	63.64
Moderate/Severe	12	36.36
NECROSIS	N	%
Absent	7	21.21
Present	26	78.79
LVSI	N	%
Absent	14	42.42
Present	19	57.58
ER	N	%
High	14	42.42
Low	19	57.58
PR	N	%
High	9	27.27
Low	24	72.73
ki67	N	%
High	23	69.70
Low	10	30.30
p53	N	%
Abnormal	21	63.64
Wild-type	12	36.36
B-CATENIN	N	%
High	27	81.82
Low	6	18.18
E-CADHERIN	N	%
High	29	87.88
Low	4	12.12
BCL-2	N	%
High	4	12.12
Low	29	87.88
CYCLIN D1	N	%
High	11	33.33
Low	22	66.67

**Table 3:** Clinical–pathological and immunohistochemical features of the study populations. Continuous variables.

	N	Minimum	Maximum	Mean	Median	SD	RSD	SEM	Normal Distr.
Age	33	53	93	74.121	76	10.532	0.1421	1.8334	0.555
FU DFS Duration	33	1	63	24.848	22	18.3969	0.7404	3.2025	0.1075
FU DOD Duration	33	1	63	27	23	18.0624	0.669	3.1443	0.0443
ER%	33	0	95	25.758	5	33.7065	1.3086	5.8675	0.0501
PR%	33	0	60	10.788	0	17.3975	1.6127	3.0285	0.0024
ki67%	33	15	90	62.576	60	19.6898	0.3147	3.4276	0.2756
p53%	33	0	100	51.697	60	43.8153	0.8475	7.6273	<0.0001
B-cat%	33	5	100	83.939	100	24.8985	0.2966	4.3343	0.0001
E-cad%	33	20	100	90	100	22.3257	0.2481	3.8864	<0.0001
Bcl-2%	33	0	100	18.182	10	24.4252	1.3434	4.2519	0.0001
Cycl-D1%	33	0	100	21.273	15	24.1535	1.1354	4.2046	0.0001

**Table 4:** Correlation table of continuous variables.

		ER%	PR%	ki67%	p53%	β-cat%	E-cad%	Bcl-2%	Cycl-D1%
	Significance Level P	0.0066	0.1466	0.6027	0.789	0.2118	0.8	0.0141	0.5912
ER%	Correlation coefficient		0.716	−0.039	−0.294	0.199	0.337	−0.213	−0.041
	Significance Level P		<0.0001	0.8288	0.0966	0.2659	0.0554	0.2334	0.8206
PR%	Correlation coefficient	0.716		−0.054	−0.236	0.14	0.343	−0.254	−0.136
	Significance Level P	<0.0001		0.7656	0.186	0.4376	0.0508	0.1544	0.4514
ki67%	Correlation coefficient	−0.039	−0.054		0.541	0.032	−0.074	−0.093	0.033
	Significance Level P	0.8288	0.7656		0.0012	0.8589	0.6839	0.6049	0.8553
p53%	Correlation coefficient	−0.294	−0.236	0.541		0.089	0.091	0.247	0.071
	Significance Level P	0.0966	0.186	0.0012		0.6217	0.6134	0.1661	0.6927
β-cat%	Correlation coefficient	0.199	0.14	0.032	0.089		0.581	0.08	−0.179
	Significance Level P	0.2659	0.4376	0.8589	0.6217		0.0004	0.6573	0.3199
E-cad%	Correlation coefficient	0.337	0.343	−0.074	0.091	0.581		0.166	−0.063
	Significance Level P	0.0554	0.0508	0.6839	0.6134	0.0004		0.3569	0.7271
Bcl-2%	Correlation coefficient	−0.213	−0.254	−0.093	0.247	0.08	0.166		0.067
	Significance Level P	0.2334	0.1544	0.6049	0.1661	0.6573	0.3569		0.7098
Cycl-D1%	Correlation coefficient	−0.041	−0.136	0.033	0.071	−0.179	−0.063	0.067	

**Table 5:** DFS continuous variables.

	Age		FU DFS Duration		ER%		PR%		ki67%	
FU_DFS	No rec	Rec	No rec	Rec	No rec	Rec	No rec	Rec	No rec	Rec
N	21.00	12.00	21.00	12.00	21.00	12.00	21.00	12.00	21.00	12.00
Mean	72.95	76.17	30.29	15.33	25.95	25.42	14.57	4.17	58.10	70.42
SD	9.85	11.79	19.86	10.59	32.39	37.38	20.02	8.75	21.48	13.56
SEM	2.15	3.40	4.33	3.06	7.07	10.79	4.37	2.53	4.69	3.91
*p*	0.30		0.03767		0.84		0.24		0.13	
	p53%		B-cat%		E-cad%		Bcl-2%		Cycl-D1%	
FU_DFS	No rec	Rec	No rec	Rec	No rec	Rec	No rec	Rec	No rec	Rec
N	21.00	12.00	21.00	12.00	21.00	12.00	21.00	12.00	21.00	12.00
Mean	49.10	56.25	82.86	85.83	87.62	94.17	8.10	35.83	21.05	21.67
SD	45.05	43.12	26.72	22.34	26.01	13.79	11.56	30.96	28.42	15.13
SEM	9.83	12.45	5.83	6.45	5.68	3.98	2.52	8.94	6.20	4.37
*p*	0.64		0.72		0.34		0.00327		0.32	

**Table 6:** OS continuous variables.

	Age		FU DOD Duration		ER%		PR%		ki67%	
FU DOD	Alive	DOD	Alive	DOD	Alive	DOD	Alive	DOD	Alive	DOD
Mean	74.88	71.75	28.56	22.125	22.2	36.875	12.24	6.25	58.6	75
SD	10.52	10.925	19.1531	14.0655	30.8923	41.6565	19.0728	10.3	19.975	13.1
SEM	2.103	3.8626	3.8306	4.9729	6.1785	14.7278	3.8146	3.63	3.995	4.63
*p*	0.69		0.50114		0.36531		0.981363		0.033036	
	p53%		B-cat%		E-cad%		Bcl-2%		Cycl-D1%	
FU DOD	Alive	DOD	Alive	DOD	Alive	DOD	Alive	DOD	Alive	DOD
Mean	48.84	60.625	82.8	87.5	88.4	95	13	34.4	20.88	22.5
SD	43.81	45.547	27.3511	15.8114	24.3977	14.1421	17.9699	35	26.7337	14.6
SEM	8.763	16.103	5.4702	5.5902	4.8795	5	3.594	12.4	5.3467	5.18
*p*	0.551		0.981935		0.32181		0.078521		0.269308	

**Table 7:** Disease-free survival univariate analysis. Histopathology and immunohistochemistry.

HISTOPATHOLOGY	*n*%		*n*%		*p*	HR	95% C.I.
Stage	NO recurrence		Recurrence				
FIGO III–IV	5	15.15	9	27.27	0.0337	3.48	1.1007 to 10.9943
FIGO I–II	16	48.48	3	9.09			
Invasion	NO recurrence		Recurrence				
Infiltrative	12	36.36	10	30.30	0.1760	2.29	0.6903 to 7.5707
Expansive	9	27.27	2	6.06			
Desmoplasia	NO recurrence		Recurrence				
Moderate/severe	6	18.18	3	9.09	0.5457	1.46	0.4283 to 4.9718
Absent/mild	15	45.45	9	27.27			
Necrosis	NO recurrence		Recurrence				
Present	15	45.45	11	33.33	0.1854	2.45	0.6509 to 9.1917
Absent	6	18.18	1	3.03			
TIL	NO recurrence		Recurrence				
Moderate/severe	8	24.24	4	12.12	0.5861	1.38	0.4319 to 4.4170
Absent/mild	13	39.39	8	24.24			
LVSI	NO recurrence		Recurrence				
Present	11	33.33	8	24.24	0.3422	1.74	0.5538 to 5.4862
Absent	10	30.30	4	12.12			
IHC	*n*%		*n*%		*p*	HR	95% C.I.
ER	NO recurrence		Recurrence				
High	10	30.3	4	12.1	0.1372	2.4	0.7563 to 7.6371
Low	11	33.3	8	24.2			
PR	NO recurrence		Recurrence				
High	8	24.2	1	3.03	0.0321	3.7	1.1180 to 12.2590
Low	13	39.4	11	33.3			
p53	NO recurrence		Recurrence		0.4098	1.6	0.5025 to 5.4046
High	12	36.4	9	27.3			
Low	9	27.3	3	9.09			
ki 67	NO recurrence		Recurrence		0.2543	2	0.5991 to 6.9390
High	13	39.4	10	30.3			
Low	8	24.2	2	6.06			
E-cadherin	NO recurrence		Recurrence				
High	18	54.5	11	33.3	0.6021	0.6	0.1197 to 3.4242
Low	3	9.09	1	3.03			
B-catenin	NO recurrence		Recurrence				
High	17	51.5	10	30.3	0.6021	0.6	0.1197 to 3.4242
Low	4	12.1	2	6.06			
Bcl-2	NO recurrence		Recurrence				
High	5	15.2	9	27.3	0.0179	8.6	1.4492 to 51.1531
Low	16	48.5	3	9.09			
Cyclin D1	NO recurrence		Recurrence				
High	6	18.2	5	15.2	0.3464	1.8	0.5197 to 6.4545
Low	15	45.5	7	21.2			

**Table 8:** Overall survival univariate analysis. Histopathology and immunohistochemistry.

HISTOPATHOLOGY		*n*%		*n*%	*p*	HR	95% C.I.
Stage	Alive		DOD				
FIGO III–IV	7	21.21	7	21.21	0.0217	5.1856	1.2721 to 21.1382
FIGO I–II	18	54.55	1	3.03			
Invasion	Alive		DOD				
Infiltrative	15	45.45	7	21.21	0.2236	2.5651	0.5627 to 11.6940
Expansive	10	30.30	1	3.03			
Desmoplasia	Alive		DOD				
Moderate/severe	6	18.18	3	9.09	0.9788	1.0203	0.2305 to 4.5174
Absent/mild	19	57.58	5	15.15			
Necrosis	Alive		DOD				
Present	18	54.55	8	24.24	0.1125	NA	NA
Absent	7	21.21	0	0.00			
TIL	Alive		DOD				
Moderate/severe	8	24.24	4	12.12	0.6737	1.3546	0.3298 to 5.5638
Absent/mild	17	51.52	4	12.12			
LVSI	Alive		DOD				
Present	13	39.39	6	18.18	0.2624	2.2415	0.5465 to 9.1940
Absent	12	36.36	2	6.06			
IHC		*n*%		*n*%	*p*	HR	95% C.I.
ER	Alive		DOD				
High	10	30.30	4	12.12	0.6187	1.4393	0.3431–6.0378
Low	15	45.45	4	12.12			
PR	Alive		DOD				
High	8	24.24	1	3.03	0.1173	3.1882	0.7473–13.6031
Low	17	51.52	7	21.21			
p53	Alive		DOD				
Abnormal	15	45.45	6	18.18	0.4592	1.7299	0.4052–7.3848
Wild-type	10	30.30	2	6.06			
ki67	Alive		DOD				
High	16	48.48	7	21.21	0.3428	2.1891	0.4337–11.0500
Low	9	27.27	1	3.03			
E-cadherin	Alive		DOD				
High	22	66.67	7	21.21	0.8738	0.8515	0.1172–6.1867
Low	3	9.09	1	3.03			
B-catenin	Alive		DOD				
High	20	60.61	7	21.21	0.9154	0.8869	0.09686–8.1207
Low	5	15.15	1	3.03			
Bcl-2	Alive		DOD				
High	8	24.24	6	18.18	0.1504	5.6206	0.5345–59.1001
Low	17	51.52	2	6.06			
Cyclin D1	Alive		DOD				
High	8	24.24	3	9.09	0.6959	0.7375	0.1603–3.3941
Low	17	51.52	5	15.15			

## Data Availability

Not applicable.

## Abbreviations

Clear cell carcinoma (CCC); disease-free survival (DFS); died of the disease (DOD); endometrial cancer (EC); estrogen receptor (ER); high-grade non-endometrioid carcinoma (HG-NEC); high-grade endometrial carcinomas (HG-ECs; immunohistochemistry (IHC); lymph–vascular space invasion (LVSI); malignant mixed Müllerian tumor (MMMT); overall survival (OS); progesterone receptor (PR); relative standard deviation (RSD); standard deviation (SD); standard error of the mean (SEM); staining index (SI); tumor lymphocytic infiltrate (TIL).
